# Edible Wild Vegetables *Urtica dioica* L. and *Aegopodium podagraria* L.–Antioxidants Affected by Processing

**DOI:** 10.3390/plants11202710

**Published:** 2022-10-14

**Authors:** Layla Engelhardt, Tobias Pöhnl, Susanne Neugart

**Affiliations:** Department of Crop Science, Division of Quality and Sensory of Plant Products, Georg-August-University Göttingen, Carl-Sprengel-Weg 1, 37075 Göttingen, Germany

**Keywords:** ABTS, antioxidant activity, chelate complex, DPPH, drying, HPLC, minerals, thermal processing, total phenolic content, storage

## Abstract

*Urtica dioica* L. and *Aegopodium podagraria* L., also known as stinging nettle and ground elder, are edible wild green vegetables rich in bioactive and antioxidant polyphenols, vitamins, and minerals. Antioxidant activity assays (TEAC-, DPPH-, and TPC-assay) in combination with HPLC measurements, to qualify and quantify their chemical compositions, were used. Firstly, the drying methods affected the antioxidant activity of further processing stages, and outcomes were dependent on the species. Secondly, cooking increased the antioxidant activity due to higher concentrations of bioactive compounds, and released bound compounds through the rupture of cell structures. Furthermore, fridge storage (3 days at 7 °C) resulted in the lowest antioxidant activity, compared to freezer storage (30 days at −20 °C). Added 5-caffeoylquinic acid (0.3 mM) led to an increased antioxidant activity, most noticeably in freeze-dried samples. Synergistic effects of 5-caffeoylquinic acid were primary found in freeze-dried samples, analyzed fresh or after storage in the fridge. Metal-chelates can lower the antioxidant activity in plant matrices. Edible wild green vegetables are rich in polyphenols and processing can even increase their concentrations to boost the potential health effects. In general, selected quantified phenolics are not solely responsible for the antioxidant activity; minerals, processing, and interactions in plant matrices also contribute decisively.

## 1. Introduction

*Urtica dioica* L. and *Aegopodium podagraria* L. are two common edible wild green vegetables, also known as stinging nettle and ground elder. They are native to the temperate zone of Western Europe and are widely distributed in other regions, such as northern Africa, Asia, and Australia [1,2]. Plants are autotrophs and need to adapt in order to survive unfavorable environmental conditions, predations, and competition for resources [3]. Wild vegetables are used to various biotic and abiotic stress factors in their natural habitats, without breeding effects. Thus, these plants developed a highly specialized morphology and synthesized a diverse array of secondary plant metabolites, such as polyphenols and vitamins [4,5], as a response to stress conditions and as protective mechanisms against reactive oxygen species (ROS), including superoxide anion radical (O_2_^•−^), singlet oxygen (^1^O_2_), hydrogen peroxide (H_2_O_2_), and the highly reactive hydroxyl radical (OH^•^) [6,7,8]. Polyphenols can be found free in the vacuole and bound to the cell walls of plants. *U. dioica* L. and *A. podagraria* L. are both rich in bioactive compounds, and especially young leaves can be used raw in salads, cooked as leafy vegetables or in soups, infused with hot water to extract tea, or for traditional medicine [9,10]. In times when food was scarce, edible wild vegetables often supplemented the daily diet. However, this valuable source of nutrients has been somewhat forgotten over the years. Due to their natural composition, wild edible green vegetables can supplement more common antioxidant sources, such as fruit and vegetables, in the human diet. By today’s knowledge, secondary metabolites are nonessential for humans, but the antioxidant activity is associated with reduced risk of cancer, cardiovascular disease, and other chronic diseases [11,12]. The human body can only absorb 5 to 10% of all consumed phenolics in the small intestine. After fermentation by the colon microbiota, bound phenolics can be released in the large intestine [13]. Post-harvest processing might enhance the bioavailability of phenolics by releasing bound phenolics. The most common postharvest preservation of wild green vegetables is drying [14]. Dehydration of herbs can be performed using different methods, affecting quality and stability of antioxidants. Freeze-drying or lyophilization is becoming more common in food applications and guarantees high quality products [15]. Traditionally, oven-drying is the most frequently used method due to its lower costs and is a common kitchen-scale method for home drying. The edible wild green vegetables *U. dioica* L. and *A. podagraria* L. are often cooked, similarly to spinach, in a small amount of water which is not drained afterwards. Prepared meals are often partly stored in fridges at 7 °C for several days or frozen at −20 °C for longer time periods, a practice that has gained importance due to closed communal catering during COVID-19. All these processes—drying, cooking, and storage—influence the concentration of bioactive compounds, their antioxidative activity, and, thus, their bioavailability to humans. Furthermore, interactions between polyphenols, vitamins, and minerals in plants can lead to additional synergistic or even antagonistic effects [16]. *U. dioica* L. and *A. podagraria* L. are rich in minerals which are essential in the human diet, e.g., iron for blood formation and calcium for bone stability. Minerals form metal chelate-complexes with other bioactive compounds, which may influence their bioactive behavior. In a previous study model, most plants with omnipresent 5-caffeoylquinic acid interacted with iron and had protective effects on other molecules, especially quercetin-3-rutinoside, which helped to avoid their decrease in concentration after thermal processing [17].

The novelty of this study is the combination of common household practices, such as drying, cooking, and storage of *A. podagraria* L. and *U. dioica* L., with targeted protection of bioactive compounds by supplemental 5-caffeoylquinic acid, to show mechanistic effects under processing conditions. The influence of processing on widely neglected edible wild green vegetables in the human diet with focus on their composition of bioactive compounds and minerals, along with their antioxidant activity, were analyzed based on the hypothesis that (1) postharvest processing has a negative effect on the antioxidant activity, (2) processing reduces the bioactive compounds concentration and influences the composition by releasing degradation products, (3) supplementation of 5-caffeoyquinic acid reduces the degradation of bioactive compounds and preserves their antioxidant activity, and (4) the ability of minerals to form chelate-complexes have an impact on the antioxidant activity.

## 2. Results

### 2.1. Composition of Bioactice Compounds

**Figure 1:**
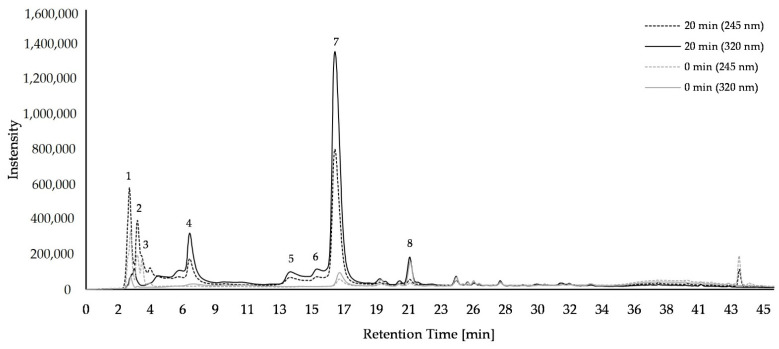
Chromatogram of ascorbic acid and phenolic compounds in *Urtica dioica* L. samples detected by HPLC-DAD after freeze-drying without storage at wavelengths of 245 nm (dotted line) and 320 nm (solid line). Cooking time of 0 min (gray) and 20 min (black); 1–3: ascorbic acid and its derivatives; 4: coumaroylglucaric acid isomer 1; 5: coumaroylglucaric acid isomer 2; 6: di-caffeoylquinic acid; 7: 5- caffeoylquinic acid; 8: caffeoylmalic acid.

In the *U. dioica* L. samples, ascorbic acid (*m/z* 175) and it derivates, protocatechuic acid glucoside (*m/z* 315), coumaroylglucaric acid isomers (*m/z* 355), di-caffeoylquinic acid (*m/z* 515), 5-caffeoylquinic acid (*m/z* 353), caffeoylmalic acid (*m/z* 295), caffeoylmalate (*m/z* 591), *p*-coumaric acid (*m/z* 163), ferulic acid (*m/z* 193), quercetin-3-rutinoside (*m/z* 609), caffeoylglucaric acid (dimer) (*m/z* 743), and feruloyl malate (*m/z* 309) were tentatively identified by HPLC-MS (Supplemental Table S1). Eight major compounds were quantified (Figure 1, Supplemental Table S2). In freeze-dried *U. dioica* L. samples, ascorbic acid and its derivates (peak 1–3) increased with cooking from 5.53 to 12.01 µg/mg sample, with added exogenous 5-caffeoylquinic acid, from 5.40 to 8.19 µg/mg sample. The concentration of coumaroylglucaric acid isomer 1 (peak 4) increased after 20 min of cooking, from 0.70 to 2.38 µg/mg sample, coumaroylglucaric acid isomer 2 (peak 5) from 0.54 to 1.86 µg/mg sample, and di-caffeoylquinic acid (peak 6) from 0.63 to 1.34 µg/mg sample, 5-caffeoylquinic acid (peak 8) increased from 3.18 to 10.30 µg/mg sample, and caffeoylmalic acid (peak 9) from 0.69 to 16.08 µg/mg sample. The addition of exogenous caffeoylquinic acid in 20 min cooked *U. dioica* L. samples led only to a decrease in ascorbic acid from 12.01 to 8.19 µg/mg sample, and to an increase of coumaroylglucaric acid isomer 1 concentration from 2.38 to 3.53 µg/mg sample. Storing of *U. dioica* L. samples in the freezer for 30 days at −20 °C had no influence on the concentrations of the compounds (Supplemental Table S2). However, samples stored in the fridge for 3 days at 7 °C, after being cooked for 20 min, showed a decrease in concentration of coumaroylglucaric acid isomer 1 and coumaroylglucaric acid isomer 2, regardless of the presence of exogenous 5-caffeoylquinic acid. In addition, the 5-caffeoylquinic acid concentration without added exogenous 5-caffeoylquinic acid decreased.

**Figure 2:**
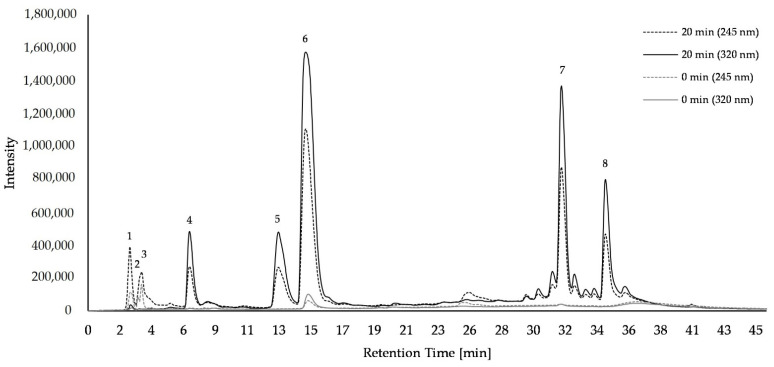
Chromatogram of ascorbic acid and phenolic compounds in *Aegopodium podagraria* L. samples detected by HPLC-DAD after freeze-drying without storage at wavelengths of 245 nm (dotted line) and 320 nm (solid line). Cooking time of 0 min (gray) and 20 min (black); 1–3: ascorbic acid and it derivates; 4: 3-caffeoylquinic acid; 5: caffeoylquinic acid dimer; 6: 5-caffeoylquinic acid; 7: di-caffeoylquinic acid; 8: kaempferol-3-glucosid.

In *A. podagraria* L. samples, ascorbic acid (*m/z* 175) and its derivates, 3-caffeoylquinic acid (*m/z* 353), 5-caffeoylquinic acid (*m/z* 353), 4-caffeoylquinic acid (*m/z* 353), coumaroylquinic acid (*m/z* 337), caffeoylquinic acid (dimer) (*m/z* 707), di-caffeoylquinic acid (*m/z* 515), caffeoylquinic acid dehydro (dimer) (*m/z* 705), quercetin-3-acetyl-glucoside (*m/z* 505), and kaempferol-3-glycosid (*m/z* 493) were tentatively identified by HPLC-MS (Supplemental Table S3). Eight major compounds were quantified (Figure 2, Supplemental Table S4). In freeze-dried *A. podagraria* L. samples, concentrations of ascorbic acid and its derivates (peak 1–3) increased with cooking from 5.03 to 5.31 µg/mg sample, and from 4.23 to 4.58 µg/mg sample in the presence of 5-caffeoylquinic acid. The concentration of 3-caffeoylquinic acid (peak 4) increased after 20 min of cooking from 0.16 to 1.72 µg/mg sample, caffeoylquinic acid dimer (peak 5) from 0.11 to 2.6 µg/mg sample, 5-caffeoylquinic acid (peak 6) from 0.79 to 12.07 µg/mg, and di-caffeoylquinic acid (peak 7) from 0.17 to 5.35 µg/mg sample. With added exogenous 5-caffeoylquinic acid, the concentration of 3-caffeoylquinic acid (peak 4) increased from 0.15 to 1.46 µg/mg sample, with caffeoylquinic acid dimer (peak 5) from 0.11 to 2.49 µg/mg sample, with 5- caffeoylquinic acid (peak 6) from 1.31 to 17.59 µg/mg sample, and with di-caffeoylquinic acid (peak 7) from 0.18 to 5.61 µg/mg sample. In 0 min cooked samples, kaempferol-3-glycosid (peak 8) was under the limit of detection regardless of exogenous 5-caffeoylquinic addition, while after 20 min of cooking the amount increased to 10.41 µg/mg or 8.0 µg/mg sample in the presence of exogenous 5-caffeoylquinic acid. Storing of *A. podagraria* L. samples in the fridge at 7 °C for 3 days or in the freezer at −20 °C for 30 days had no influence on the concentrations of the compounds (Supplemental Table S4).

### 2.2. Minerals

All analyzed minerals showed higher concentrations in *U. dioica* L. samples than in *A. podagraria* L. samples (Table 1). Only the concentration of potassium (K) was higher in *A. podagraria* L. samples than in *U. dioica* L.

### 2.3. Effects of Processing on The Antioxidant Activity in U. dioica L. 

#### 2.3.1. Effect of The Drying Method on the Antioxidant Activity of *U. dioica* L.

**Figure 3:**
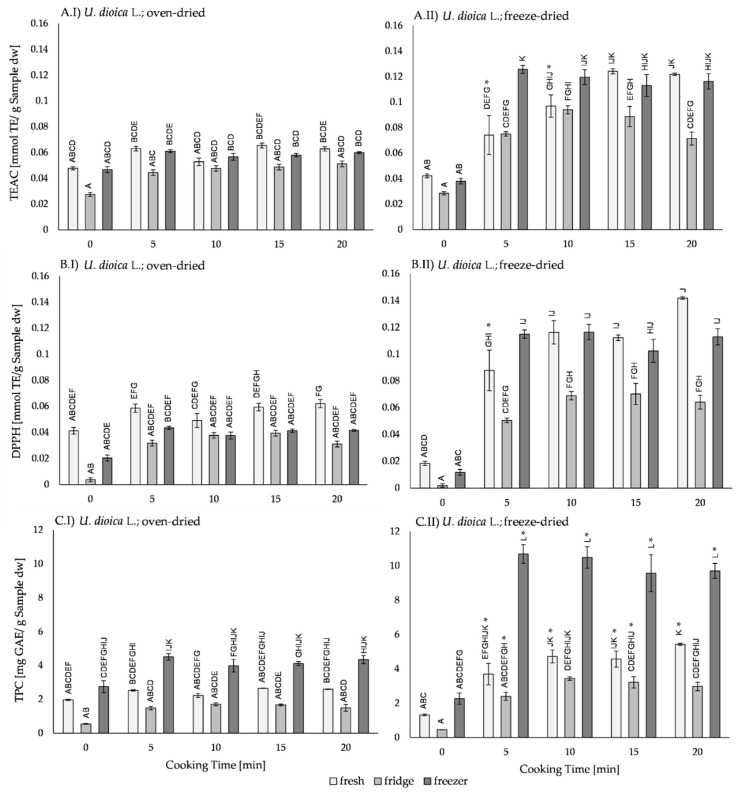
Antioxidant activity of (I) oven-dried and (II) freeze-dried uncooked (0 min) and cooked (5, 10, 15, and 20 min) *Urtica dioica* L. samples measured by (**A**) TEAC-, (**B**) DPPH-, and (**C**) TPC assays. Fresh samples (light grey) and samples stored after cooking in the fridge (7 °C for 3 days; medium grey) or in the freezer (−20 °C for 30 days; dark grey) were compared. Bars represent mean ± standard error (SE). Significant differences (*p* ≤ 0.05 by Tukey’s HSD test) are marked with uppercase letters. Differences between samples with and without addition of 5-caffeoylquinic acid are marked with an asterisk (*).

Comparing the freeze-dried and the oven-dried *U. dioica* L. samples, in all test assays (Figure 3), no differences in the antioxidant activity of 0 min cooked samples were found, regardless of the storage type. In 5 to 20 min cooked samples, samples which were freeze-dried fresh and stored in the freezer had higher antioxidant activity compared to oven-dried samples. Solely in fresh samples, after 5 min of cooking, the antioxidant activity was not different from the 0 min cooked samples. In all fridge-stored samples, no difference in the antioxidant activity between the cooked freeze-dried and oven-dried samples was found. Only in the TEAC assay (Figure 3A), after 10 and 15 min of cooking, higher antioxidant activity in freeze-dried samples were detected. Samples with the addition of exogenous 5-caffeoylquinic acid (Supplemental Figure S1), showed a similar pattern.

#### 2.3.2. Effect of Thermal Processing on The Antioxidant Activity of *U. dioica* L.

Thermal processing had no influence on the antioxidant activity of oven-dried samples (Figure 3I) within the same storage type. Cooked freeze-dried samples (Figure 3II) showed higher antioxidant activity compared to 0 min cooked samples. Furthermore, cooking time between 5 and 20 min had no influence on the antioxidant activity. With the addition of exogenous 5-caffeoylquinic acid (Supplemental Figure S1), the TEAC assay revealed higher antioxidant activity in all cooked samples, except for those stored in the fridge after 5 min of cooking. The DPPH assay showed higher antioxidant activity after 15 min and 20 min of cooking only in the fridge-stored samples, and the TPC assay revealed higher antioxidant activity in fridge-stored samples after 10 min to 20 min of cooking than in the 0 min cooked sample. After 5 min of cooking, the antioxidant activity did not differ from the 0 min cooked samples.

#### 2.3.3. Effect of Storage on the Antioxidant Activity of *U. dioica* L.

The three storage types—fresh, fridge, and freezer—showed no differences in the antioxidant activity in the oven-dried samples (Figure 3I), regardless of thermal processing time. In the TPC assay (Figure 3CI), freezer-stored samples had higher antioxidant activity than fridge-stored samples, except after 10 min of cooking. In 0 min cooked freeze-dried samples (Figure 3II) no differences in the antioxidant activity were found. In freeze-dried samples cooked for 5 to 20 min, fridge-stored samples showed lower antioxidant activity than fresh and/or freezer-stored samples. Exceptions were found in the TPC assay (Figure 3CII), where the freezer-stored samples were higher in their antioxidant activity than fresh and fridge-stored samples. With exogenous 5-caffeoylquinic acid addition (Supplemental Figure S1), in the TEAC assay, the oven-dried, 0 min cooked, fresh samples had higher antioxidant activity than fridge-stored samples. In freeze-dried samples, the fridge-stored samples had lower antioxidant activity compared to the fresh and frozen samples.

### 2.4. Effects of Processing on the Antioxidant Activity in A. podagraria L.

#### 2.4.1. Effect of Drying on the Antioxidant Activity of *A. podagraria* L.

**Figure 4:**
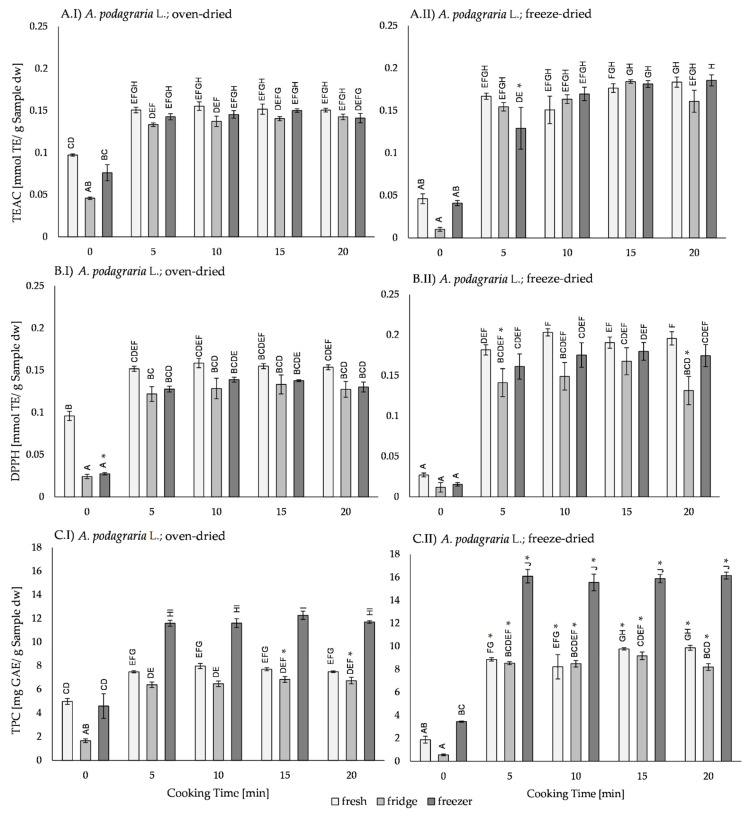
Antioxidant activity of (I) oven-dried and (II) freeze dried uncooked (0 min) and cooked (5, 10, 15, and 20 min) *Aegopodium podagraria* L. samples measured by (**A**) TEAC-, (**B**) DPPH-, and (**C**) TPC assays. Fresh samples (light grey) and samples stored after cooking in the fridge (7 °C for 3 days; medium grey) or in the freezer (−20 °C for 30 days; dark grey) were compared. Bars represent mean ± standard error (SE). Significant differences (*p* ≤ 0.05 by Tukey’s HSD test) are marked with upper case letters. Differences between samples with and without addition of 5-caffeoylquinic acid are marked with an asterisk (*).

Comparing the oven-dried and the freeze-dried *A. podagraria* L. samples (Figure 4), the oven-dried and 0 min cooked samples had higher antioxidant activity in fresh samples, while after storage in the fridge and the freezer, no differences in the antioxidant activity in freeze-dried samples were detected. In cooked samples, no differences in antioxidant activity between drying methods were found. In the TEAC assay (Figure 4A), freezer-stored samples had higher antioxidant activity in freeze-dried samples after 20 min of cooking. Furthermore, in the TPC assay (Figure 4C), after 5, 10, and 20 min of cooking, the antioxidant activity was higher in the freezer-stored and freeze-dried samples. After adding exogenous 5-caffeoylquinic acid (Supplemental Figure S2), the antioxidant activity of most freeze-dried samples was higher in cooked samples than in oven-dried samples.

#### 2.4.2. Effect of Thermal Processing on the Antioxidant Activity of *A. podagraria* L.

Cooked samples had higher antioxidant activity than 0 min cooked samples, which were treated with the same storage and drying method. Cooking between 5 and 20 min did not influence the antioxidant activity. With added exogenous 5-caffeoylquinic acid (Supplemental Figure S2), the TEAC and DPPH assay oven-dried samples, showed higher antioxidant activity only after 15 and 20 min of cooking. 

#### 2.4.3. Effect of Storage on the Antioxidant Activity of *A. podagraria* L.

In most analyzed samples, the storage method did not influence the antioxidant activity. Exceptions were found in oven-dried samples, fridge-stored, and 0 min cooked samples, which showed lower antioxidant activity than fresh and/or freezer-stored samples. It was conspicuous that in the TPC assay, without exogenous 5-caffeoylquinic acid addition, the freezer-stored and cooked samples had higher antioxidant activity than the fresh or fridge-stored samples.

### 2.5. Synergistic and Antagonistic Effects on the Antioxidant Activity by Exogenous 5-Caffeoylquinic Addition

Addition of exogenous 5-caffeoylquinic acid had no effect on the total antioxidant activity of *A. podagraria* L. (Supplemental Figure S2) but tends to increase. In the TPC assay only, exogenous 5-caffeoylquinic acid led to an increase in antioxidant activity in freeze-dried fresh and fridge-stored samples, while it led to a decrease in freezer-stored samples. In the TEAC assay, the addition of exogenous 5-caffeoylquinic acid to freeze-dried *U. dioica* L. samples (Supplemental Figure S1) increased the antioxidant activity in the 5 min and 10 min cooked fresh samples. In the DPPH assay, the antioxidant activity of only the 5 min cooked fresh samples increased. TPC assay results showed higher antioxidant activity in fresh and fridge-stored samples, but lower antioxidant activity in freezer-stored samples after addition of exogenous 5-caffeoylquinic acid.

**Figure 5:**
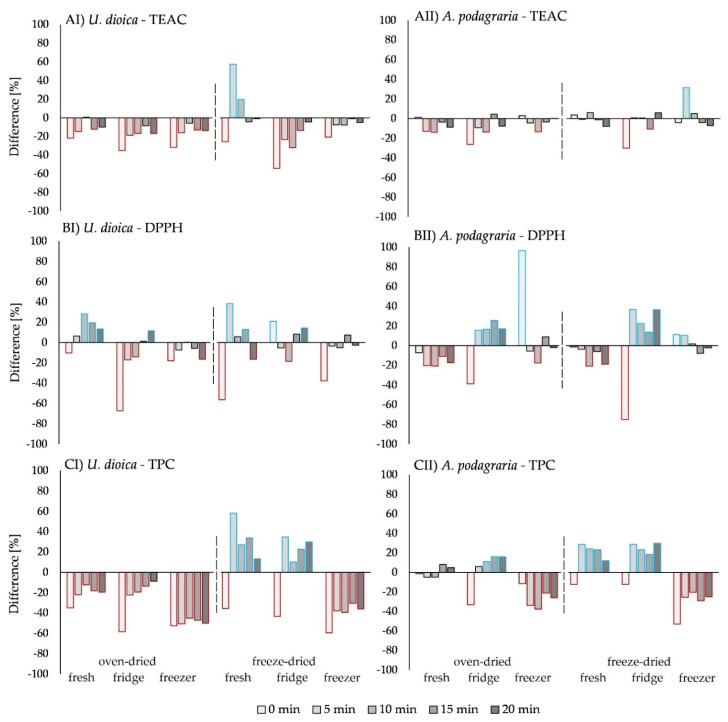
Difference in percent [%] of the experimental antioxidant activity from the calculated sum of the plant sample’s individual antioxidant activities and the 5-caffeoylquinic acid of I) *Urtica dioica* L. and II) *Aegopodium podagraria* L. using (**A**) TEAC, (**B**) DPPH, and (**C**) TPC assays. Synergistic effects over +10% are marked with blue frames, antagonistic effects over −10% are marked with red frames.

Experimentally obtained antioxidant activity (Supplemental Figures S1 and S2) was compared with theoretical antioxidant activity values, calculated by the sum of the measured antioxidant activity of pure plant samples and the 5-caffeoylquinic acid separately. Mainly antagonistic effects were found, meaning that the measured antioxidant activity was lower than the theoretical value with 5-caffeoylquinic acid addition, especially in 0 min cooked samples (Figure 5). Synergistic effects were found in *U. dioica* L. samples, whereas in freeze-dried and fresh samples, a synergism which decreased with increasing cooking time (negative time–dose–response) was revealed. In the DPPH assay, oven-dried fresh samples also showed a negative time–dose–response. However, in freeze-dried, fridge-stored samples, the DPPH and TPC assay showed an increasing synergism with increasing cooking time (positive time–dose–response). In samples of *A. podagraria* L., synergistic effects were found in 0 min cooked freezer-stored samples by the DPPH assay. In oven-dried fridge-stored samples, a positive time–dose–response was found, while in the TPC assay, a negative time–dose–response was found in freeze dried fresh and fridge-stored samples. 

## 3. Discussion

### 3.1. Influence of Drying Methods on Antioxidant Activity

Drying is a process that ensures a microbiologically stable and durable product, due to moisture loss [18]. However, all drying methods, including oven-drying or freeze-drying, have an influence on the plant compounds and thus on the antioxidant activity and compound stability. In *U. dioica* L. samples, the antioxidant activity of 0 min cooked samples did not differ between oven-dried and freeze-dried, while *A. podagraria* L. samples had higher antioxidant activity in oven-dried fresh samples than in the freeze-dried fresh samples. A reason for this could be the different morphology and phenol composition of *A. podagraria* L., and the fact that oven-drying allows metabolic processes to continue until full dryness [14], whereas these processes are stopped due to the freezing procedure before freeze-drying. García et al. [19] found that in 0 min cooked *U. dioica* L., the leaves had higher antioxidant activity in freeze-dried than in oven-dried samples, measured by TPC and DPPH assay. Compared to a lack of differences in the antioxidant activity detected in the results, differences may be explained by the drying temperature. In this study, oven-drying was performed at only 40 °C, while García et al. [19] dried their samples at 105 °C. Drying at moderate temperatures, around 40 °C, does not negatively influence the antioxidant activity of 0 min cooked samples in this study. The higher antioxidant activity in cooked freeze-dried *U. dioica* L. and *A. podagraria* L. samples, here found solely in the TPC assay, are based on the species and drying method in combination with temperature. This could be due to matrix-bound molecules released during the cooking process [20]. Oven-drying also leads to a degradation of bioactive compounds due to oxygen exposure and a longer phase of moisture during drying. Freeze-drying is known to be better for preserving bioactive compounds due to low temperature, minimum exposure to oxygen, and a lack of water for reactions [21]. The tested species, especially the freeze-dried *U. dioica* L. samples, seem to profit from the preserving properties. Prior studies have already shown that the taxonomic family also had an influence on the degradation ration of phenolic compounds. Sledz et al. [22] showed that after microwave-convective drying, plants belonging to the family of Apiaceae, including *A. podagraria* L., had lower degradation ratios compared to Brassicaceae and Lamiaceae. Mirhosseini et al. [23] suggested that based on the morphology, the optimal drying method needs to be chosen for the purpose of retaining most of the valuable bioactive compounds responsible for the antioxidant activity. Another reason for the differences between drying methods may be the enzymes in complex plant matrices. The process of freeze-drying can deactivate enzymes such as polyphenol oxidase [24], while during oven-drying, the enzymes are active for a longer period of time due to the slower moisture loss and oxygen exposure [25]. Most enzymes are temperature sensitive; optimal conditions are between 30 °C and 45 °C, matching the temperature of 40 °C during oven-drying. Consequently, the enzyme activities were only stopped by moisture lost during drying. The longer duration of enzyme activity during oven-drying may have influenced the bioactive compounds’ concentration and composition. After samples were remoisturized with water, some of the enzymes may have been reactivated [25]; thus, in 0 min cooked samples stored in the fridge, enzymes can be active and tend to cause lower antioxidant activity.

### 3.2. Thermal Processing

Heat can influence the concentration and composition, and thus the antioxidant activity, of bioactive compounds in vegetables, herbs [25,26,27], and of pure compound standards [17]. The measured compounds in *U. dioica* L. are comparable to those found in previous studies [19,28]. For *A. podagraria* L. the data for phenolic composition are rare. Hydroxycinnamic acids, flavonoid glycosides, and derivates of quercetin and kaempferol were mentioned [2,29]. Previously, the focus in *A. podagraria* L. was set mainly on essential oils [30]. An increasing concentration of the measured main compounds in samples of *U. dioica* L. and *A. podagraria* L. after 20 min of cooking goes along with the increase in the antioxidant activity. Notably, not one specific phenolic compound was responsible for the increase in antioxidant activity; it is, rather, the sum of the phenolic compounds concentration that led to higher antioxidant activity. Similar results were found previously in plantains [31], eggplants [32], potatoes [33], and sweet potatoes [34]. Most phenolic compounds are not free in the plant matrices; they are covalently bound to the cell wall. Heat energy weakens the still-intact cell matrices and releases the cell wall-bound phenolics [20]. The appearance of coumaroylglucaric acids and caffeoylglucaric acid (dimer) in *U. dioica* L. samples after cooking can be explained by the release of these cell wall-bound substances, because, in the vacuole and cell wall matrix, plant cells contain estimated 20% to 60% of bound phenolics [13]. The increase in the polyphenol concentrations in *U. dioica* L. and *A. podagraria* L. samples after cooking is based on the disbandment of the carboxyl group ester bound from the hydroxyl groups of cell wall substances [35,36]. An increase in secondary plant metabolites following thermal processing, such as in *U. dioica* L. and *A. podagraria* L., was also shown for *cis*-lycopene in tomatoes [36], which indicates that the bounding energies may also play a role. Some bounds come apart earlier; others need more thermal energy. Many bioactive compounds are known to be heat-sensitive. A well known and powerful antioxidant, ascorbic acid, decreased in concentration during thermal treatment in pure compound standard mixtures [17] as well as in green leafy vegetables such as *Brassica carinata* A. Braun and *Brassica oleracea* L. [37]. A decrease in phenolic compounds occurred after processing, mainly due to deglycosylation and degradation [38,39], which can have lower, higher, or the same antioxidant activity as the precursory bioactive compounds derived from [40]. During thermal processing, bound phenolics were released from the matrix, which also leads to an increase in quantified polyphenol concentrations and detection of further polyphenols, such as kaemperol-3-glucoside in *A. podagraria* L. samples after 20 min of cooking. In oven-dried *U. dioica* L. samples without exogenous 5-caffeoylquinic acid addition, no differences were found between the cooked and uncooked samples. This may be due to the plant morphology as well as the oven-drying process, wherein leaves were exposed to oxygen and prolonged enzymatic degradation occurred due to the leaves remaining moist for a longer period of time. Since the plant tissue and the cooking water were analyzed together, the leaching effect of water-soluble bioactive compounds to the water had no influence on this experiment. 

### 3.3. Storage

Typical household forms of food storage, namely eating the prepared food right after cooking, storing leftovers in the fridge at 7 °C for around 3 days, or placing them in the freezer at −20 °C for 30 days were modelled. Based on the Van ‘t Hoff equation, lower temperatures reduce the reaction speed. Consequently, in freezer-stored samples, the degradation is reduced. The higher antioxidant activity in freezer-stored samples compared to fridge-stored samples was caused by the formation of ice crystals during the freezing process, which can rupture the cell structure [41] and release antioxidant active compounds [42]. In freeze-dried samples, fridge-stored samples showed or tended to have lower antioxidant activity compared to the fresh or freezer-stored samples. This can be explained by oxidation processes in the aqueous phase. After storage at 10 °C for 1–3 days, the antioxidant activity of kale and carrot juice decreased, which was detected by the TPC assay [43]. Meanwhile, in fresh-cut spinach stored for 7 days at 10 °C, no changes in antioxidant activity were found [44]. Contrary to the results of the antioxidant activity assays, the amount of quantified bioactive compounds coumaroylglucaric acid isomer 1, coumaroylglucaric acid isomer 2 and 5-caffeoylquinic acid in freeze-dried samples of *U. dioica* L. was lower after storage in the fridge. In oven-dried *A. podagraria* L. samples, only di-caffeoylquinic acid decreased in 0 min cooked and fridge-stored samples. In fact, chemically different phenolics and the plant matrices in which they are bound have different contributions to the cooking and storage behavior. In *A. podagraria,* kaemperol-3-glucoside was only detected after thermal processing (Supplemental Table S2) and did not change in concentration after storage in the fridge or freezer. In a study on berries, which were cooked and cold stored (−20 °C) for 3 to 9 months, the concentration of myricetin and kaempferol decreased more than the concentration of quercetin [45]. Matrix effects have not been studied in detail due to their complexity but will lead to more robust results.

### 3.4. Addition of Exogenous 5-caffeoylquinic acid

Exogenous added 5-caffeoylquinic acid led, in most of the analyzed samples of *U. dioica* L. and *A. podagraria* L., to an increase in antioxidant activity. This is most likely due to the antioxidative activity of 5-caffeoylquinic acid itself [17]. Addition of 5-caffeoylquinic acid should increase the antioxidant activity in the TEAC assay by 0.014 mmol TE/g sample, in the DPPH assay by 0.013 mmol TE/ g sample, and in the TPC assay by 1.38 mg GAE/g sample [17]. The synergistic and antagonistic effects indicated an interaction of 5-caffeoylquinnic acid with the plant matrix. In particular, 5-caffeoylquinic acid in combination with iron showed increased antioxidant activity in a previous study [17]. It seems that the plant species had an influence on the synergistic effects, while in *U. dioica* L., predominantly freeze-dried fresh and fridge-stored samples showed synergistic effects. In *A. podagraria* L., this phenomenon was shifted to fridge- and freezer-stored samples. The synergistic effects may be explained by interactions which are active after thermal processing. Moreover, the ratios and the composition of polyphenols in the plants seem to play a role. Mitra et al. [46] demonstrated the complexity of the synergetic effects of polyphenols, as well as the fact that science is still just beginning to understand these processes. In cooked, freezer-stored *U. dioica* L. samples, as well as in *A. podagraria* L. samples, a decrease in antioxidant activity was detected regardless of drying method when measured by TPC assay. There are different factors which may influence these results, one being the working mechanism of the TPC assay. Structural features, such as the number and position of OH groups, are essential for the antioxidative activity, while the differentiation between phenolic acids and different subgroups of flavonoids is less meaningful. Platzer et al. [47] found that the highest reducing capacity is achieved by a maximum number of OH groups, qualifying for the Bors criteria. The Bors criteria are defined by the following: (I) catechol group on the B-ring, (II) 2,3 double bond and 4-oxo group on the C-ring, and (III) -OH groups at position 3 and 5 on the A- and C-rings and 4-oxo group on the C-ring [47]. However, 5-caffeoylquinic acid fulfills only one Bors criterion, and therefore has medium reducing capacity. This may not be the case in the TEAC and DPPH assays, based on mechanisms and reaction sides of the radicals. Other factors might be the phytochemical composition and the properties during processing. The higher antioxidant activity of the freeze-dried samples without exogenous 5-caffeoylquinic acid addition may be explained by interactions between polyphenols, minerals and other bioactive compounds in the plant matrix when compared to the other types of storage, or to the low values with exogenous added 5-caffeoylquinic acid. Cell wall-bound phenolics released by heat and ice crystals might react with the free 5-caffeoylquinic acid to non-bioactive compounds. 

### 3.5. Interactions of Minerals Contained in the Plant Matrix

Minerals and their concentrations may also play an important role in antioxidant activity, based on their ability to form complexes with other bioactive compounds. The measured mineral content differs from previously reported concentrations [48]. This may be due to different environmental factors and different mineral content in the soils [49]. Minerals and polyphenols, such as 5-caffeoylquinic acid [50] and quercetin-3-rutinoside [51], are known for building complexes. In particular, compounds that contain two or more of the functional groups in their structure, such as -OH, -SH, -COOH, -PO_3_H_2_, C O, -NR_2_, -S-, and -O-, are able to chelate with metal ions [52]. Approximately twice the amount of calcium (Ca), iron (Fe), magnesium (Mg), phosphorus (P), and sulfur (S) were found in *U. dioica* L. compared to *A. podagraria* L. Thus, chelate complexes may be a factor next to the phenolic composition contributing to the lower antioxidant activity in *U. dioica* L. Furthermore, Zhou and Yu [53] showed in 38 commonly consumed vegetables that there is a correlation between chelating activity of Fe^2+^ and TPC value, arguing that lower TPC values indicate lower Fe^2+^ chelation. Therefore, the higher iron concentration in *U. dioica* L. in the TPC assay may also contribute to lower antioxidant activity compared to *A. podagraria* L., due to chelation. Certain metal ions, when combined or mixed with an organic ligand, can lead to an increase in the antioxidant activity [51]. Additional factors, including pH value [50], available polyphenols, and other plant nutrients such as other minerals or proteins [54], have an impact on both the antioxidant and bioactive activity of the plant samples. The analyzed wild edible plants *U. dioica* L. and *A. podagraria* L. seem to be a rich source of minerals in the human diet, even if there are various possible interactions with their polyphenols, which are not fully understood so far.

## 4. Materials and Methods

### 4.1. Chemicals

ABTS^●+^ (2,2’-azino-bis (3-ethylbenzothiazoline-6-sulfonic acid) diammonium salt) (≥ 98%) was obtained from Sigma-Aldrich (Steinheim, Germany), DPPH^●^ (2,2-diphenyl-1-picrylhydrazyl) radical (95%) and Trolox^®^ (97%) were obtained from Thermo Fisher (Kandel, Germany). Folin–Ciocalteu phenol reagent was purchased from Merck (Darmstadt, Germany). Methanol (MeOH; HPLC grade), acetonitrile (ACN; HPLC grade), glacial acetic acid (100%, p.a.), sodium acetate trihydrate (≥ 99.5% p.a.), gallic acid monohydrate (≥ 99%), potassium persulfate (≥ 99%), rutin trihydrate (working standard), chlorogenic acid (working standard), and L- (+)-ascorbic acid (working standard) were purchased from Carl Roth (Karlsruhe, Germany). Ultrapure water (H_2_O) was purified by arium^®^ pro obtained from Satorius (Göttingen, Germany).

### 4.2. Plant Material

In spring 2020, plant samples of 414.05 g *Urtica dioica* L. and 428.16 g *Aegopodium podagraria* L. were collected in forest areas around Göttingen, Germany. The aerial portions of the plants were collected and brought to the laboratory within two hours. Only the leaves of the young plants were used. In order to eliminate biotic and abiotic factors of single plants, each species was pooled to obtain a homogenous sample mixture. The plants were identified based on the descriptions by Schmeil-Fitschen [55]. From each species, one aliquot was freeze-dried for 96 h, and another was oven-dried at 40 °C for 48 h. Dried plant material was ground to powder with agate balls.

### 4.3. Procesing and Storage

In closed microtubes, 20 mg of plant powder filled up with 500 µL H_2_O or with a 0.3 mM 5-caffeoylquinic acid solution were boiled for 0, 5, 10, 15, and 20 min. In order to stop the heating process immediately, samples were subsequently put on ice. After each cooking treatment, one sample was analyzed immediately (fresh), one was stored for 3 days in the fridge (7 °C) and another was stored for 30 days in the freezer (−20 °C). Samples were extracted in three extraction steps. In the first step, 500 µL MeOH was added to the sample with the cooking water, shaken in a Thermo-Mixer at 20 °C for 40 min at 1400 rpm, then centrifuged at 10,000 rpm (rotor radius 10 cm) for 10 min. The supernatant was transferred to another micro tube. In the second and third step, 400 µL of MeOH/H_2_O (1:1, *v*/*v*) was added to the pellet of the pervious extraction step, followed by shaking in the Thermo-Mixer at 20 °C for 15 min at 1400 rpm and centrifugation. The combined supernatants were vacuum-dried and filled up again with 1000 µL H_2_O. Before use, samples were filtered with a cellulose acetate Spin-X^®^ Centrifuge Tube Filter (0.22 µm).

### 4.4. Photometric Measurements

Antioxidant activity and total reducing activity were measured photometrically using a high-throughput method in 96-well plates (Synergy™ HTX Multi-Mode Microplate Reader, BioTek Instruments, Winooski, VT, USA). Three independent biological and two technical replicates were measured for each sample.

#### 4.4.1. Total Phenolic Content (TPC)

The commonly known TPC assay with Folin–Ciocalteu reagent, which detects the “total reducing activity” of bioactive compounds, was applied as described earlier [17]. All wells were prefilled with 10 µL Folin–Ciocalteu reagent, 50 µL sample, and 100 µL Na_2_CO_3_ were added. Incubation took place at 37 °C (±0.2 °C) and orbital shaking at medium speed (237 cpm, 4 mm) for 14 min. After 1 min delay, the UV-absorbance was measured at 736 nm. Results were expressed as gallic acid equivalents [mg GAE/g sample (dry weight)], using a standard curve ranging from 5.97 to 59.7 µg gallic acid (R^2^ > 0.98).

#### 4.4.2. Trolox Equivalent Antioxidant Capacity (TEAC)

The TEAC assay was measured by ABTS-radical reduction as described previously [17]. A stock solution with 9.6 mg ABTS and 1.66 mg potassium persulfate, filled up with H_2_O up to 25 mL, was prepared and incubated in the dark at room temperature for 12 to 16 h. For the final working solution, 5 mL stock solution filled up to 25 mL with 100% MeOH. In a 96-well plate, 10 µL samples were mixed with 150 µL of the TEAC working solution. After 5 min of incubation time, 1 min orbital shaking at medium speed, and 1 min delay, the absorbance was measured at 734 nm. Results were expressed as Trolox Equivalents [mmol TE/g sample (dry weight)], using a standard curve ranging from 0.025 to 0.7 mM Trolox (R^2^ > 0.98).

#### 4.4.3. 2,2-diphenyl-1-picrylhydrazyl (DPPH)

The DPPH assay was modified for better comparison to the TEAC assay results, as described previously [17]. For the DPPH working solution, 7.88 mg DPPH was filled up to 100 mL with 100% MeOH and incubated in the dark at room temperature for 2 h. Briefly, 20 µL of the sample was mixed with 180 µL of the DPPH working solution and incubated in the dark for 28 min at room temperature. After 1 min orbital shaking by medium speed and 1 min delay, the absorbance was measured at a wavelength of 515 nm. Results were expressed as Trolox equivalents [mmol TE/g sample (dry weight)], using a standard curve ranging from 0.025 to 0.7 mM Trolox (R^2^ > 0.98).

### 4.5. HPLC

Quantification and identification of the known substances and their changes during processing in the plant samples were assessed using a Shimadzu Prominence 20 high performance liquid chromatography (HPLC) system equipped with a refrigerated SIL-20AC HT autosampler, a CTO-10AS VP column oven, a DGU-20A5 degasser, an LC-20 AT liquid chromatograph quaternary pump, an SPD-M20A diode array detector (DAD), and a CBM-20A communication bus module. A Supelco Ascentis^®^ Express F5 150 × 3.0 mm, 5 µm, with a Supelco Guard column 5.0 × 3.0 mm, 5 µm, and a 0.2-micron SST Frits for UltraLine was also used. The column temperature was set to 30 °C. The mobile phase consisted of Eluent A (1% acetic acid ) and Eluent B (100% ACN). The separation was achieved using the following gradient: 0–7 min, 5% B; 7–30 min, 5–20% B; 30–49.5 min 20–90% B; 49.5–52 min 90% B, 52–52.7 min 90–5% B; 52.7–59 min 5% B. The flow rate was 0.3 mL/min and the injection volume was 30 µL. Standard curves of 5-caffeoylquinic acid (0.564–0.042 mM; R^2^ > 0.99; 320 nm), ascorbic acid (0.568–0.043 mM; R^2^ > 0.99; 245 nm) and quercetin-3-rutinoside (0.614–0.046 mM; R^2^ > 0.99; 360 nm) were used for quantification of the phenolic compounds (Supplemental Tables S2 and S4). The limit of detection (LOD; factor 3.3) and quantification (LOQ; factor 10) were calculated with the standard error of the intercepts and the slopes of the calibration curves. For identification of the compounds, samples were measured in a negative ion mode by an Aglient 6460 HPLC-QQQ equipped with an electrospray ionization (ESI) interface, applied to perform mass spectrometry, at the division of Molecular Phytopathology and Mycotoxin Research of the Georg-August-Universität Göttingen, Germany. Ion source parameters were set as follows: capillary voltage = +4000/−4000 V, gas temperature = 350 °C, gas flow rate = 13 L/min, and nebulizer = 60 psi. The fragmentation voltage (135 V) was applied for collision-induced dissociation.

### 4.6. Mineral Analysis

Plant samples were prepared by microwave-assisted digestion. Briefly, 100 mg of dried plant sample were weighed. 4 mL HNO_3_ and 2 mL 30% H_2_O_2_ were added and placed in a microwave at 200 °C and 15 bar pressure for 80 min. Afterward, they were filled up to 25 mL with H_2_O. The measurements were made with an ICAP 7000 Series ICP Spectrometer from Thermo Scientific by the Albrecht-Haller-Institute of Georg-August-University Göttingen, Germany.

### 4.7. Statistical Analysis

For biostatistics tests and presenting data results, Microsoft Excel 2016 (Microsoft, Redmond, USA) and R Statistics (version 3.6.3, Holding the Windsock, 2020), with the R packages emmeans [56] and multcomp [57], were used. Inferential statistics for assessing and linking treatments were assessed by using analysis of variance (ANOVA) and a post hoc Tukey’s HSD test.

## 5. Conclusions

Despite some species-specific differences, some basic statements can be made. A combination of freeze-drying and boiling leads to the highest antioxidant activity in the samples. Overall, the duration of cooking between 5 and 20 min has only a minor influence on the antioxidant activity. For *U. dioica* L., consumption immediately after cooking or after storage at −20 °C for up to 30 days is preferable to consumption after storage at 7 °C for 3 days. For *A. podagraria* L., storage seems to play a minor role. Protection of phenolic compounds by 5-caffeoylquinic acid appears to be effective, if at all, only for freshly cooked samples or samples stored at 7 °C for 3 days. For samples stored at −20 °C for 30 days, a negative effect of additional 5-caffeoylquinic acid administration on antioxidant activity was observed. In *U. dioica* L., freeze-dried samples showed higher concentrations of phenolic compounds than oven-dried samples, except for ascorbic acid, which was found in high concentrations in oven-dried samples. In contrast, in *A. podagraria* L., the cooked and freeze-dried samples showed higher concentrations of phenolic compounds. Cooking leads to higher concentrations of phenolic compounds in the samples in both species studied. In *U. dioica* L., only slight changes in the concentrations of phenolic compounds are found. Reductions occur in the samples stored at 7 °C for 3 days. In *A. podagraria* L., the effects of storage on phenolic compounds are even smaller. In *U. dioica* L., the addition of 5-caffeoylquinic acid mainly increases the glucaric acid derivatives in cooked samples, especially in freshly cooked samples and samples stored in the refrigerator. In *A. podagraria* L., such an effect is almost completely absent. But the addition of antioxidants not necessarily lead to synergetic or antagonistic effects in this study.

Food matrices are complex, and many reactions can occur, resulting in various effects. The plant morphology and its chemical composition need to be considered in postharvest processing. In some species, such as in *U. dioica* L. and *A. podagraria* L., cooking released bound phenolics from the cell walls, which led to higher concentrations of phenolics before consumption. This indicates potentially increased bioavailability to the human body. In the future, the gained knowledge needs to be transferred to other plant species, as well as from this theoretical based method to practical food preparation. In our daily diets, plant matrices often interact with matrices of other plant- or animal-based products. Thus, complex interactions between food matrices may lead to unintentional degradation of valuable antioxidants. This needs to be investigated in complex food matrices instead of solely mechanistic approaches in order to give real insight into nutritive value.

## Figures and Tables

**Table 1:** Mineral content in mg per 100 g fresh weight (fw) of collected *Urtica dioica* L. and *Aegopodium podagraria* L. samples (n = 3), and the differences between them.

Minerals	*U. dioica* L. [mg/100g fw]	*A. podagraria* L. [mg/ 100g fw]	Difference *
Al	0.867 ± 0.022	0.629 ± 0.032	+ 1.4
Ca	396.546 ± 1.586	144.217 ± 1.293	+ 2.7
Cu	0.379 ± 0.038	0.317 ± 0.052	+ 1.2
Fe	2.084 ± 0.013	1.370 ± 0.017	+ 1.5
K	490.703 ± 3.407	584.154 ± 4.047	- 1.2
Mg	50.041 ± 0.357	34.927 ± 0.253	+ 1.4
Mn	0.842 ± 0.006	0.692 ± 0.009	+ 1.2
Na	1.797 ± 0.478	1.427 ± 0.374	+ 1.3
P	107.967 ± 0.441	48.638 ± 0.451	+ 2.2
S	87.197 ± 0.480	28.878 ± 0.074	+ 3.0
Zn	0.721 ± 0.013	0.657 ± 0.131	+ 1.1

* Difference = *U. dioica* L. mineral concentration/*A. podagraria* L. mineral concentration.

## Data Availability

The data presented in this study are available on request from the corresponding author.

## Supplementary Materials

The following supporting information can be downloaded at: https://www.mdpi.com/article/10.3390/plants11202710/s1, Figure S1. Antioxidant activity of (I) oven-dried and (II) freeze dried uncooked (0 min) and cooked (5, 10, 15, and 20 min) from *Urtica dioica* L. samples with added 5 caffeoylquinic acid (CQA) measured by (A) TEAC, (B) DPPH-, and (C) TPC assay. Figure S2. Antioxidant activity of (I) oven-dried and (II) freeze-dried uncooked (0 min) and cooked (5, 10, 15, and 20 min) from *Aegopodium podagraria* L. samples with added 5 caffeoylquinic acid (CQA) measured by (A) TEAC-, (B) DPPH-, and (C) TPC assay. Table S1. Mass spectra, retention times, and absorbance maxima of phenolic compounds and ascorbic acid derivatives tentatively identified in *Urtica dioica* L., by HPLC QQQ and HPLC DAD. Table S2. Concentrations of selected phenolic compounds and ascorbic acid derivatives [µg/mg sample] in *Urtica dioica* L. uncooked (0 min) and after 20 min of cooking. Table S3. Mass spectra, retention times, and absorbance maxima of phenolic compounds and ascorbic acid derivatives tentatively identified in *Aegopodium podagraria* L., by HPLC QQQ and HPLC DAD. Table S4. Concentrations of selected phenolic compounds and ascorbic acid derivatives [µg/mg sample] in *Aegopodium podagraria* L. uncooked (0 min) and after 20 min of cooking. Refs. [58,59,60,61] are cited in supplementary material file.
