# Supplementary material for: Edible Wild Vegetables Urtica dioica L. and Aegopodium podagraria L.–Antioxidants Affected by Processing
Source: Plants (Basel). 2022 Oct 14;11(20):2710. doi: 10.3390/plants11202710 (PMC9610176; doi:10.3390/plants11202710)
Supplement: Supplementary file 1 [file plants-11-02710-s001.zip › plants-1884935-supplementary.pdf]

Supplementary

Table S1. Concentrations of selected phenolic compounds and ascorbic acid derivatives [µg/mg sample] in *Urtica dioica* L. uncooked (0 min) and after 20 min of cooking. Significant differences within one compound were calculated by ANOVA and Tukey HSD ($p \leq 0.05$). AsAD: ascorbic acid and its derivatives, CGA: coumaroylglucaric acid (isomer 1/isomer 2), diCQA: di-caffeoylquinic acid , CQA: 5-caffeoylquinic acid, CMA: caffeoylmalic acid.

	AsAD[1]	CGA 1[2]	CGA 2[2]	diCQA[2]	CQA[2]	CMA[2]	Total	AsAD*[1]	CGA 1[2]	CGA 2[2]	diCQA[2]	CQA[2]	CMA[2]	Total
	**freeze-dried**							**oven-dried**						
fresh 0 min	5.53 *a*	0.70 *ab*	0.54 *a*	0.63 *abc*	3.18 *a*	0.69 *ad*	11.27	13.25 *ab*	0.55 *a*	0.24 *ab*	0.61 *bcd*	3.46 *bcd*	0.60 *ac*	18.71
fresh 0 min + CQA	5.40 *a*	0.50 *ab*	0.18 *a*	0.30 *ab*	1.43 *a*	0.57 *ac*	8.38	11.36 *a*	0.41 *a*	0.36 *ab*	0.44 *ac*	1.92 *ab*	0.37 *ab*	14.86
fresh 20 min	12.01 *d*	2.38 *c*	1.86 *cd*	1.34 *cd*	10.30 *c*	0.94 *ad*	28.83	16.08 *ab*	0.71 *ab*	0.38 *ab*	0.25 *ab*	4.10 *d*	0.54 *ac*	22.06
fresh 20 min + CQA	8.19 *ac*	3.53 *d*	2.14 *d*	1.80 *d*	12.13 *c*	1.21 *d*	29.00	15.89 *ab*	1.84 *d*	0.92 *c*	1.08 *e*	4.20 *d*	0.61 *ac*	24.54
fridge 0 min	6.55 *ac*	0.32 *ab*	0.14 *a*	0.07 *a*	0.17 *a*	0.39 *ab*	7.64	17.03 *ab*	0.18 *a*	0.11 *a*	0.11 *a*	0.15 *a*	0.35 *a*	17.93
fridge 0 min + CQA	7.41 *ac*	0.21 *a*	0.00 *a*	0.13 *a*	0.13 *a*	0.35 *a*	8.23	18.52 *b*	0.34 *a*	0.11 *a*	0.11 *a*	0.14 *a*	0.42 *ab*	19.64
fridge 20 min	9.21 *cd*	1.19 *b*	2.94 *b*	0.51 *abc*	4.05 *ab*	0.77 *ad*	18.67	15.14 *ab*	0.99 *ad*	0.24 *ab*	0.21 *a*	2.67 *bcd*	0.50 *ac*	19.75
fridge 20 min + CQA	9.62 *cd*	2.25 *c*	1.30 *bc*	1.04 *bd*	8.92 *bc*	1.06 *cd*	24.19	17.15 *ab*	1.65 *cd*	0.85 *c*	0.76 *ce*	3.80 *cd*	0.83 *c*	25.04
freezer 0 min	5.67 *ab*	0.61 *ab*	0.12 *a*	0.15 *ab*	0.62 *a*	0.56 *ac*	7.73	13.11 *ab*	0.63 *ab*	0.36 *ab*	0.49 *acd*	1.97 *ac*	0.62 *ac*	17.18
freezer 0 min + CQA	5.75 *ab*	0.50 *ab*	0.12 *a*	0.20 *ab*	0.97 *a*	0.60 *ad*	8.14	14.21 *ab*	0.85 *abc*	0.25 *ab*	0.78 *de*	2.37 *bcd*	0.43 *ab*	18.89
freezer 20 min	8.89 *bcd*	2.18 *c*	1.80 *cd*	1.31 *cd*	9.30 *c*	1.0 *bcd*	24.48	16.77 *ab*	0.91 *abc*	0.58 *bc*	0.41 *ab*	3.95 *d*	0.72 *bc*	23.34
freezer 20 min + CQA	7.52 *ac*	2.64 *cd*	1.73 *cd*	1.37 *cd*	8.95 *bc*	1.04 *cd*	23.25	14.30 *ab*	1.50 *bd*	0.62 *bc*	0.76 *ce*	3.18 *bcd*	0.61 *ac*	20.97

[1] calibration with ascorbic acid; [2] calibration with 5-caffeoylquinic acid; *NA* = no peaks were detected.

Table S2. Concentrations of selected phenolic compounds and ascorbic acid derivatives [µg/mg sample] in *Aegopodium podagraria* L. uncooked (0 min) and after 20 min of cooking. Significant differences within one compound were calculated by ANOVA and Tukey HSD ($p \leq 0.05$). AsAD: ascorbic acid derivatives, 3-CQA: 3-caffeoylquinic acid, CQA: 5-caffeoylquinic acid, CQAdim: caffeoylquinic acid dimer, diCQA: di-caffeoylquinic acid, K3G: kaempferol-3-glucosid.

	**freeze-dried**							**oven-dried**						
	**AsAD[1]**	**3-CQA[2]**	**CQAdim[2]**	**5-CQA[2]**	**diCQA[2]**	**K3G[3]**	**Total**	**AsAD[1]**	**3-CQA[2]**	**CQAdim[2]**	**5-CQA[2]**	**diCQA[2]**	**K3G[3]**	**Total**
fresh<br>0 min	5.03<br>*ac*	0.16<br>*a*	0.11<br>*a*	0.79<br>*a*	0.17<br>*a*	*NA*	6.26	5.63<br>*a*	0.33<br>*a*	0.23<br>*a*	7.21<br>*ab*	4.03<br>*b*	*NA*	17.43
fresh<br>0 min + CQA	4.23<br>*ab*	0.15<br>*a*	0.11<br>*a*	1.31<br>*a*	0.18<br>*a*	*NA*	5.98	5.62<br>*a*	0.28<br>*a*	0.35<br>*a*	10.01<br>*b*	3.6<br>*b*	*NA*	19.86
fresh<br>20 min	5.31<br>*ac*	1.72<br>*b*	2.60<br>*b*	12.07<br>*b*	5.35<br>*b*	10.41<br>*ab*	37.46	6.06<br>*a*	1.94<br>*b*	2.29<br>*b*	11.01<br>*b*	3.59<br>*b*	10.83<br>*b*	24.89
fresh<br>20 min + CQA	4.58<br>*ac*	1.46<br>*b*	2.49<br>*b*	15.63<br>*b*	5.61<br>*b*	8.00<br>*ab*	37.77	5.98<br>*a*	2.23<br>*b*	2.01<br>*b*	9.32<br>*b*	3.95<br>*b*	5.74<br>*ab*	29.23
fridge<br>0 min	4.85<br>*ac*	0.11<br>*a*	0.10<br>*a*	0.10<br>*a*	0.11<br>*a*	*NA*	5.27	5.59<br>*a*	0.13<br>*a*	0.11<br>*a*	0.17<br>*a*	0.15<br>*a*	*NA*	6.15
fridge<br>0 min + CQA	5.94<br>*ac*	0.10<br>*a*	0.10<br>*a*	0.17<br>*a*	0.17<br>*a*	*NA*	6.48	5.85<br>*a*	0.12<br>*a*	0.10<br>*a*	0.25<br>*a*	0.2<br>*a*	*NA*	6.52
fridge<br>20 min	7.22<br>*c*	1.96<br>*b*	3.12<br>*b*	14.35<br>*b*	5.5<br>*b*	10.07<br>*ab*	42.22	6.42<br>*a*	1.93<br>*b*	2.37<br>*b*	9.24<br>*b*	3.42<br>*b*	6.22<br>*ab*	29.60
fridge<br>20 min + CQA	7.77<br>*c*	2.03<br>*b*	3.04<br>*b*	18.89<br>*b*	7.51<br>*b*	10.07<br>*ab*	49.31	6.37<br>*a*	2.54<br>*b*	2.39<br>*b*	11.22<br>*b*	4.07<br>*b*	6.23<br>*ab*	32.82
freezer<br>0 min	3.93<br>*ab*	0.17<br>*a*	0.11<br>*a*	0.83<br>*a*	0.16<br>*a*	*NA*	5.20	5.47<br>*a*	0.34<br>*a*	0.28<br>*a*	6.96<br>*ab*	3.48<br>*b*	*NA*	16.53
freezer<br>0 min + CQA	3.42<br>*a*	0.13<br>*a*	0.11<br>*a*	0.95<br>*a*	0.14<br>*a*	*NA*	4.75	5.30<br>*a*	0.26<br>*a*	0.25<br>*a*	7.69<br>*ab*	1.71<br>*ab*	*NA*	15.21
freezer<br>20 min	6.82<br>*bc*	2.34<br>*b*	3.57<br>*b*	19.82<br>*b*	6.78<br>*b*	13.23<br>*b*	52.56	6.00<br>*a*	2.37<br>*b*	2.23<br>*b*	9.69<br>*b*	3.56<br>*b*	6.94<br>*ab*	30.79
freezer<br>20 min + CQA	4.29<br>*ac*	1.72<br>*b*	1.96<br>*b*	14.00<br>*b*	5.62<br>*b*	2.05<br>*a*	29.64	5.93<br>*a*	1.97<br>*b*	1.90<br>*b*	7.61<br>*ab*	2.92<br>*ab*	5.55<br>*a*	25.88

[1] calibration with ascorbic acid; [2] calibration with 5-caffeoylquinic acid; [3] calibration with quercetin-3-rutinosid; *NA* = no peaks were detected.

Table S3. Mass spectra, retention times, and absorbance maxima of phenolic compounds and ascorbic acid derivatives tentatively identified in *Urtica dioica* L., by HPLC-QQQ and HPLC-DAD.

**MS**		**MS²**	**Ret. time (Fig. 1)**	**λ_{max} (nm)**	**Peak no. (Fig. 1)**	**Compound**	**Reference**
MW	[M-H]⁻	**[M-H]⁻**					
176	175	**	2.9; 3.3; 3.6	210, 245	1-3	ascorbic acid derivates	standard
316	315	153, 109			*	protocatechuic acid glucoside	[58]
356	355	209, 191, 85	6.6	323, 222, 410, 471	4	coumaroylglucaric acid isomer	
356	355	209, 191, 85	13.5	221, 324, 471	5	coumaroylglucaric acid isomer	
516	515	353, 191	14.6	221, 324, 409	6	di-caffeoylquinic acid	[58]
254	353	191, 179, 173, 135, **	16.4	210, 324, 243	7	5-caffeoylquinic acid	standard
296	295	151, 133	21.3	313, 223, 410, 471	8	caffeoylmalic acid	[28,59]
592	591	295, 133			*	caffeoylmalate	[28]
164	163	119			*	*p*-coumaric acid	[58,28,60]
194	193	179, 149, 135, 119			*	ferulic acid	[28,60]
610	609	301, **			*	quercetin-3-rutinoside	standard, [28]
744	743	209, 371			*	caffeoylglucaric acid (dimer)	[61]
310	309	291, 171			*	feruloyl malate	[28]

* Peaks were tentatively identified in the MS-experiment only and were not part of the quantitative analysis by HPLC-DAD. ** Identified only by spiking the sample with standards.

Table S4. Mass spectra, retention times, and absorbance maxima of phenolic compounds and ascorbic acid derivatives tentatively identified in *Aegopodium podagraria* L., by HPLC-QQQ and HPLC-DAD.

MS		MS^2	Ret. time	λ_{max} (nm)	Peak no. (Fig. 2)	Compound	Reference
MW	$[M-H]^-$	$[M-H]^-$					
176	175	**	2.9; 3.3; 3.6	210, 245	1-3	ascorbic acid derivatives	standard
354	353	191, 179, 135, **	14.6	210, 324, 243	6	5-caffeoylquinic acid	standard, [2]
354	353	191, 179, 135, **	6.7	324, 221, 523, 471	4	3-caffeoylquinic acid	standard, [2,58]
354	353	191, 179, 135, **			*	4-caffeoylquinic acid	standard, [2]
338	337	191			*	coumaroylquinic acid	[2,30]
706	705	513, 339			*	caffeoylquinic acid dehydro (dimer)	[2,61]
516	515	353, 191, 179, 155	31.5	325, 243, 224	7	di-caffeoylquinic acid	[2,58]
708	707	353, 191	12.8	325, 222, 241, 524	5	caffeoylquinic acid (dimer)	[61]
506	505	301			*	quercetin-3-acetyl-glucoside	[61]
494	493	447, 101	34.7	327, 243, 223, 471	8	kaempferol-3-glucoside	[2]

* Peaks were tentatively identified in the MS-experiment only and were not part of the quantitative analysis by HPLC-DAD. ** Identified only by spiking the sample with standards.

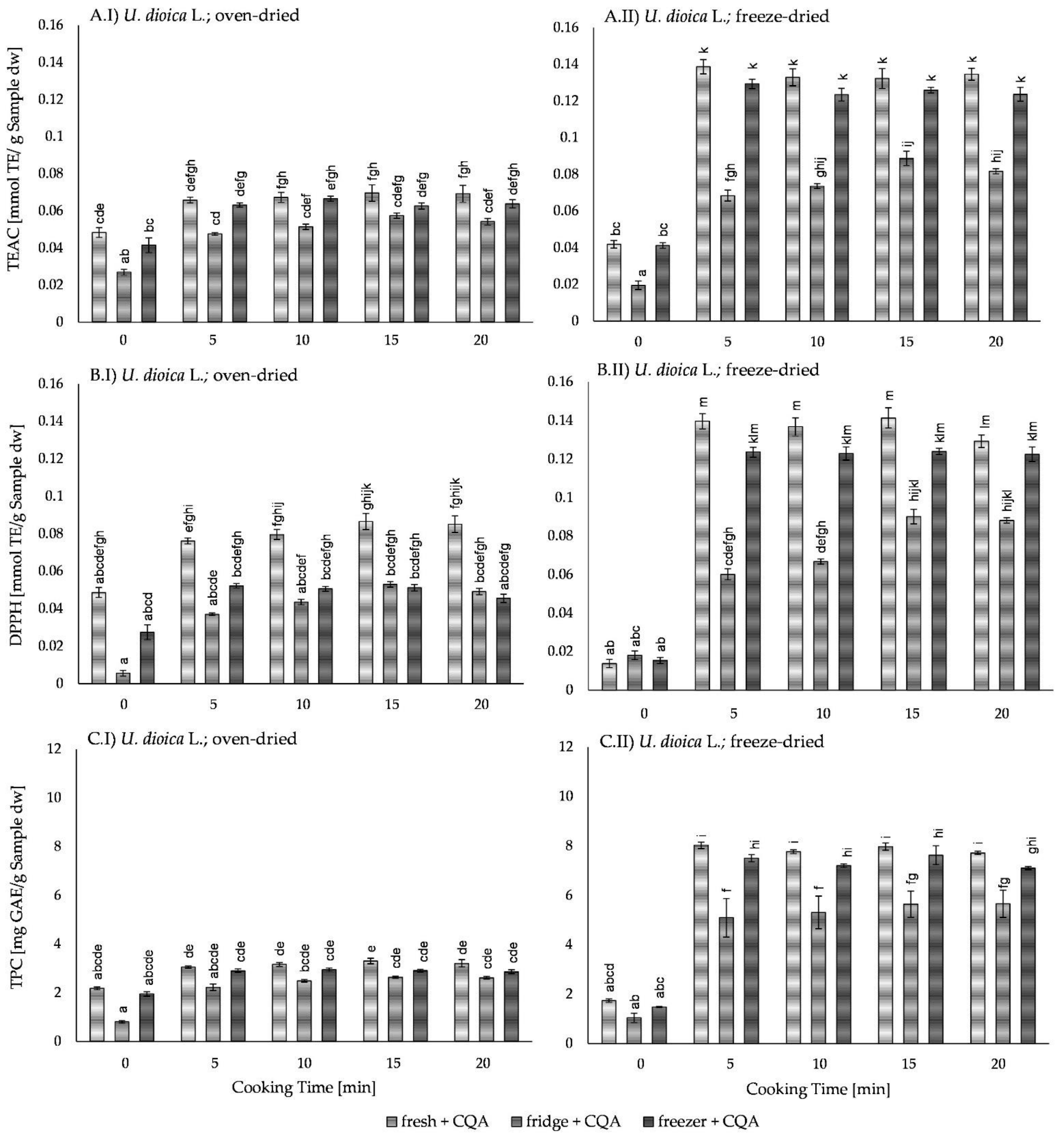

Figure S1. Antioxidant activity of (I) oven-dried and (II) freeze dried uncooked (0 min) and cooked (5, 10, 15, and 20 min) from *Urtica dioica* L. samples with added 5-caffeoylquinic acid (CQA) measured by (A) TEAC-, (B) DPPH- and (C) TPC assay. Fresh samples (light grey), samples stored after cooking in the fridge (7°C for 3 days; medium grey) and samples stored in the freezer (-20°C for 30 days; dark grey) were compared. Bars represent mean ± standard error (SE). Significant differences ($p \leq 0.05$ by Tukey's HSD test) marked with lower case letters.

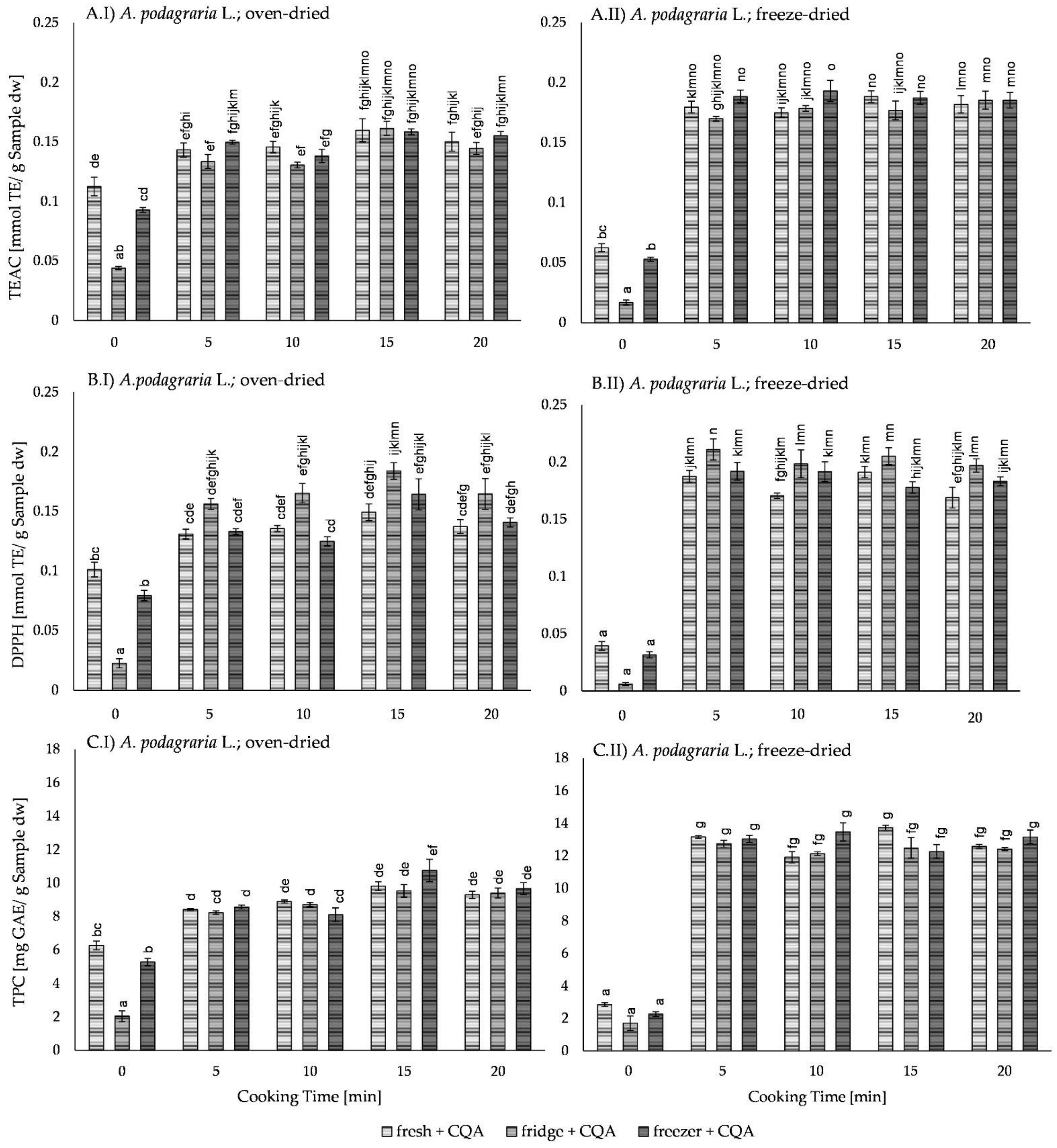

Figure S2. Antioxidant activity of (I) oven-dried and (II) freeze dried uncooked (0 min) and cooked (5, 10, 15, and 20 min) from *Aegopodium podagraria* L. samples with added 5-caffeoylquinic acid (CQA) measured by (A) TEAC-, (B) DPPH- and (C) TPC assay. Fresh samples (light grey), samples stored after cooking in the fridge (7°C for 3 days; medium grey) and samples stored in the freezer (-20°C for 30 days; dark grey) were compared. Bars represent mean ± standard error (SE). Significant differences ($p \leq 0.05$ by Tukey's HSD test) marked with lower case letters.
